# Matrix Metalloproteinase 3 Promotes Cellular Anti-Dengue Virus Response via Interaction with Transcription Factor NFκB in Cell Nucleus

**DOI:** 10.1371/journal.pone.0084748

**Published:** 2014-01-08

**Authors:** Xiangyang Zuo, Wen Pan, Tingting Feng, Xiaohong Shi, Jianfeng Dai

**Affiliations:** Institute of Biology and Medical Sciences, Jiangsu Key Laboratory of Infection and Immunity, Soochow University, Suzhou City, Jiangsu Province, People's Republic of China; Nanyang Technological University, Singapore

## Abstract

Dengue virus (DENV), the causative agent of human Dengue hemorrhagic fever, is a mosquito-borne virus of immense global health importance. Characterization of cellular factors promoting or inhibiting DENV infection is important for understanding the mechanism of DENV infection. In this report, MMP3 (stromelysin-1), a secretory endopeptidase that degrades extracellular matrices, has been shown promoting cellular antiviral response against DENV infection. Quantitative RT-PCR and Western Blot showed that the expression of MMP3 was upregulated in DENV-infected RAW264.7 cells. The intracellular viral loads were significantly higher in MMP3 silenced cells compared with controls. The expression level of selective anti-viral cytokines were decreased in MMP3 siRNA treated cells, and the transcription factor activity of NFκB was significantly impaired upon MMP3 silencing during DENV infection. Further, we found that MMP3 moved to cell nucleus upon DENV infection and colocalized with NFκB P65 in nucleus. Co-immunoprecipitation analysis suggested that MMP3 directly interacted with NFκB in nucleus during DENV infection and the C-terminal hemopexin-like domain of MMP3 was required for the interaction. This study suggested a novel role of MMP3 in nucleus during viral infection and provided new evidence for MMPs in immunomodulation.

## Introduction

Dengue virus (DENV), a member of the mosquito-borne flavivirus family, is an enveloped virus with a single-stranded positive sense RNA genome. DENV circulates in tropical and subtropical regions of the world and about 50 millions of DENV infection are estimated to occur annually worldwide[1]. Dengue hemorrhagic fever, the severe form of DENV infections, can cause serious haemorrhage, sudden drop in blood pressure (shock) and even death [1], [2]. Currently, there is neither approved vaccine nor any antiviral drug available for prevention and treatment of dengue [1], [3]. Much effort is needed to explore the host anti-viral mechanisms for control of DENV infection and vaccine development.

Cells release a large number of anti-viral cytokines including interferons (IFNs) upon DENV infection [3], [4]. These cytokines rapidly activate JAK-STAT signaling pathway, and the transcription factor complexes (STATs) start the transcription of many antiviral genes including hundreds of IFN-induced genes (ISGs) and many other regulatory effectors[5], [6], [7]. Although the JAK-STAT signaling pathway is well known to play a crucial role in antiviral innate immune response, the specific functions of the distinct downstream effectors remain largely unknown [5], [8]. In depth studies are needed to characterize the function of downstream signaling molecules of JAK-STAT during DENV infection. Matrix metalloproteinase 3 (MMP3), a downstream effector molecule of JAK-STAT signaling pathway [9], was found upregulated in macrophage upon DENV infection during our screening. However, the role of MMP3 during virus infection is unclear.

MMPs are zinc-dependent endopeptidases and comprise a large family of enzymes with different abilities to degrade specific extracellular matrix (ECM) components[10], [11]. MMPs are traditionally considered responsible for the remodeling and turnover of ECM in physiological processes such as angiogenesis, wound healing, embryogenesis, and morphogenesis as well as in pathological states including cancers, myocardial infarction, fibrotic disorders, rheumatism and osteoarthritis[10], [11]. But recently, MMPs have been shown to function in innate immunity and inflammation probably by modulating cytokine/chemokine activity and other proteins[12], [13]. For example, MMP9 deficiency results in enhanced allergen-induced airway inflammation [14]. Our previous study suggested that MMP9 facilitates West Nile Virus entry into the brain by enhancing the permeability of blood brain barrier[15]. MMP3, also known as stromelysin-1, has been associated with pathogenesis of neurodegenerative disease including Alzheimer's disease (AD) and Parkinson's disease (PD)[16], [17]. Several studies suggested an important role of MMP3 as a signaling molecule in the neuronal apoptotic process as well as neuroinflammation[16], [17], [18], [19], [20]. MMP3 is implicated to involve in activating microglia in the apoptotic neuronal cells and can influence the expression of pro-inflammatory cytokines or iNOS induced by LPS in microglia[21]. But to date, the role of MMP3 during virus infection remains largely unknown. In the present study, we elaborate the relevance of MMP3 to DENV infection using *in vitro* model.

## Materials and Methods

### Virus, Cells and Infection

DENV-2 virus (DENV New Guinea C stain) was propagated in mosquito C6/36 cells as described[22]. RAW264.7, MEF, 293T, A549 and Vero E6 cells were obtained from American Type Culture Collection (ATCC) and used for DENV-2 infection at a MOI = 1, unless specified. Cells were maintained in proper mediums supplemented with 10% fetal bovine serum, 1% penicillin/streptomycin in humidified air containing 5% CO_2_ at 37°C according to ATCC's guidelines.

Tissue culture infective dose (TCID_50_) of DENV-2 in infected cell supernatants was determined according to standard protocols[22] on Vero cells.

### Plasmids and Antibodies

Recombinant plasmids for expression of mouse and human MMP3 were constructed using standard protocols by inserting mouse or human MMP3 ORF into vector pCMV; pdsRed or pcDNA-flag. Truncated human MMP3 proteins encoding MMP3 amino acid residues 1–100, 100–265, 265–477, 100–477 were cloned into pcDNA-flag via a PCR-based method. Recombinant plasmids for expression of human NFκB (GFP-RelA (p65) and pCMV-p50) were obtained from Addgene. Reporter plasmids NFκB-Luc, AP1-Luc and pRL-TK were purchased from Clontech and used for dual luciferase reporter assays.

Primary antibodies Rabbit anti-MMP3 Polyclonal (Proteintech); Mouse anti-Dengue virus (Santa Cruz); Rabbit anti-NFκB (p105/p50) (EPTIOMICS); Rat anti-GFP (Biolegend); Rat anti-DYKDDDK (flag) (Biolegend); Actin Monoclonal antibody (Proteintech) and secondary antibodies HRP-Donkey anti-rabbit IgG; HRP-Goat anti-rat IgG(Biolegend); HRP-Goat anti-Mouse IgG(Biotech); FITC labeled Donkey anti-mouse IgG and TRITC labeled Donkey anti-rabbit IgG (Jackson ImmunoResearch) were used in this study.

### RNA interference and Quantitative RT-PCR (q-PCR)

For transiently silencing MMP3, cells were transfected with siRNA targeting mouse or human MMP3 gene (or Negative Control siRNA) via Lipofectamine 2000 (Invitrogen) transfection or electroporation (for RAW264.7). The siRNA sequences for mouse and human MMP3 gene were: 5-AAUUCCAACUGCGAAGAUCCACUGA-3 and 5-GAGUUUGACCCAAAUGCAAAGAAAG-3, respectively. RNAi efficiency was confirmed by Quantitative RT-PCR and Western Blot.

mRNA expression level for mouse gene *Mmp3*, *Ifnb1*(Interferon beta 1), *Il6* (Interleukin-6), *Cxcl1* (Chemokine (C-X-C motif) ligand 1), *Cxcl2* (Chemokine (C-X-C motif) ligand 2),*Ccl5* (Chemokine (C-C motif) ligand 5), and *Ccr5* (C-C chemokine receptor type 5) were measured by SYBRGreen based q-PCR using gene specific primers (Applied Biosystems) and normalized with mouse beta actin gene. The intracellular viral loads, in terms of the transcript levels of the DENV-2 envelop gene (E), were quantified by quantitative PCR and normalized to mouse beta actin gene. The sequences of q-PCR primers for DENV-2 E gene are 5-CATTCCAAGTGAGAATCTCTTTGTCA-3 and 5-CAGATCTCTGATGAATAACCAACG-3.

### Enzyme-Linked Immunosorbent Assay (ELISA)

RAW264.7 cells were transfected with N.C. or mouse *Mmp3* siRNA via electroporation and at 24 hrs post-transfection were infected with DENV-2 for another 24 hrs. Mouse TNF-α and IL-6 in cell supernatants were measured using ELISA kits (Biolegend Systems) following the manufacturers' instructions.

### Immunofluorescence microscopy

To analyze the subcellular distribution of MMP3 upon DENV-2 infection, 293T cells were transfected with pcDNA-flag-MMP3 using Lipofectamine 2000 reagent and infected with DENV-2 at a MOI = 1 at 24 hrs post-transfection. 48 hrs after infection, cells were fixed in 1% PFA. DENV envelope protein and MMP3 were probed with primary antibodies (anti-DENV E mouse IgG and anti-MMP3 rabbit IgG) and stained with FITC labeled anti-mouse IgG and TRITC labeled anti-rabbit IgG, respectively. Cells were then examined by confocal microscope.

For co-localization study, 293T cells were co-transfected with pdsRed-MMP3 and GFP-RelA (p65) and infected (or not infected) with DENV-2. 48 hrs post-infection, cells were fixed in 1% PFA, and cell nuclei were stained with DAPI. The localization of RelA (p65) and MMP3 inside cells were then examined by confocal microscope.

### Luciferase reporter assays

For luciferase reporter assays, 70% confluent HEK293T (or A549) cells were transfected with 10 ng of pRL-TK reporter (herpes simplex virus thymidine kinase promoter driving renilla luciferase; internal control), 100 ng of NFκB or AP1 luciferase reporter (firefly luciferase; experimental reporter) plasmid, and either 100 ng of recombinant expressing plasmids (Vector, MMP3-flag or MMP3 fragments) or 50 nM siRNAs (N.C. or MMP3 siRNA). At 24 h post-transfection, cells were infected with DENV-2 at a MOI = 1. The luciferase activity was measured after another 24 hrs, using a Promega Dual Glow kit according to the manufacturer's instructions.

### Co-Immunoprecipitation and Western Blot

To analyze the potential interaction between MMP3 and NFκB, co-Immunoprecipitation were performed in 293T cells co-transfected with flag-MMP3 (or truncated MMP3), GFP-RelA(p65) and P50. Whole cell extracts (200 µg) were prepared from transfected cells and incubated for 2 hrs at 4°C with 50 ul of ANTI-FLAG M2-Agarose Affinity Gel (Sigma). Agarose were washed five times and the bound proteins were eluted by boiling for 3 min in SDS protein loading buffer. Then the eluted samples were subjected to SDS-PAGE and transferred onto a PVDF membrane. The potential signal of RelA (p65) or P50 were detected by immunoblotting with anti-GFP or anti-P50 antibodies.

To analyze whether there is an interaction between MMP3 and NFκB in nucleus, the nuclear proteins from transfected cells were prepared using NE-PER Nuclear and Cytoplasmic Extraction kit (PIERCE) according to the manufacturer's instructions. The Co-Immunoprecipitation and Western Blot were performed as described above.

### Statistical Analysis

Statistical significances were calculated with an unpaired two-tailed Student's t- test using Prism 5 software (GraphPad).

## Results

### *Mmp3* is upregulated upon DENV infection

**Figure 1:**
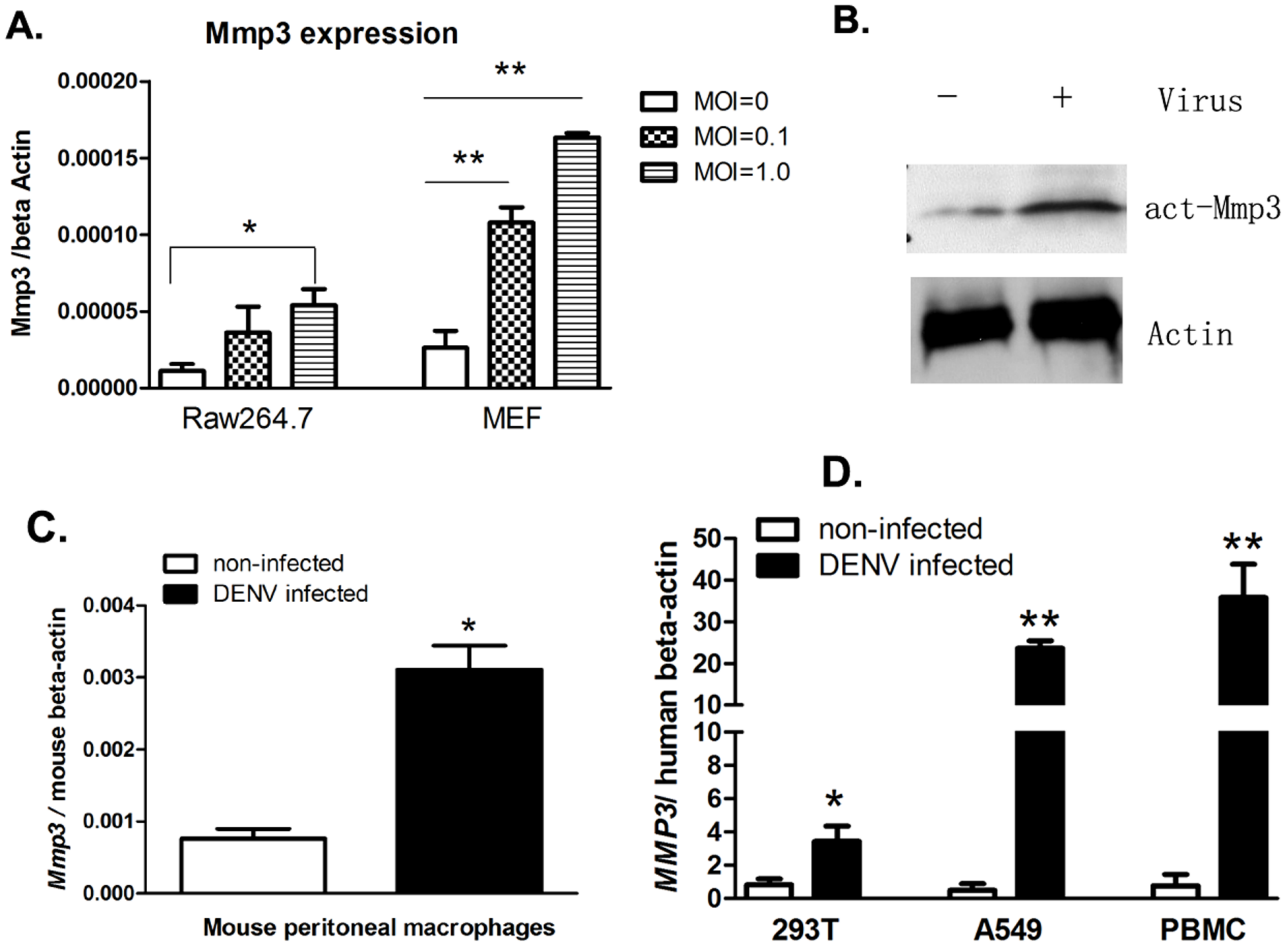
*Mmp3* is upregulated upon DENV infection. A. Mouse *Mmp3* expression in DENV-2 infected or uninfected RAW264.7 and MEF cells were analyzed by quantitative RT-PCR (qRT-PCR), and normalized to mouse beta actin gene. Results are expressed as the mean + the SEM. * p<0.05 and ** p<0.01 (*t*-test). B. The protein level of mouse MMP3 was increased in DENV infected RAW264.7 cells (MOI = 1.0, 48 hrs post infection) compared with uninfected cells. C. Mouse *Mmp3* mRNA expression in DENV-2 infected or uninfected mouse peritoneal macrophages. (MOI = 1.0, 48 hrs post infection). D. Human *MMP3* mRNA expression increased in human PBMC, 293T and A549 cells upon DENV infection. (MOI = 1.0, 48 hrs post infection, respectively). Representative results from at least 3 independent experiments.

Mouse macrophage cell line RAW264.7 can be infected by DENV, and served as an *in vitro* model for the study of host innate immune response to DENV[23]. By using a quantitative RT-PCR (qRT-PCR) based small cDNA array (SABiosciences, Frederick, MD), we measured the expression profile of genes from JAK-STAT signaling pathway in DENV infected RAW264.7 cells (Additional File 1 of Ref [23]). As a downstream effector gene of STATs, *Mmp3* was upregulated 3.68-fold in DENV infected cells in comparison to the uninfected controls. To further verify the upregulation of *Mmp3* during DENV infection, RAW264.7 and MEF (mouse embryonic fibroblast) cells were infected with different dose of DENV to analyze the expression of *Mmp3*. The *Mmp3* transcription was shown upregulated upon DENV infection in a virus dose dependent manner as determined by quantitative RT-PCR (qRT-PCR). Western Blot confirmed the upregulation of MMP3 protein in RAW264.7 cells upon DENV infection (Figure 1, A and B). MMP3 were also significantly upregulated in mouse primary peritoneal macrophage, as well as in human peripheral blood mononuclear cell (PBMC) and epithelial cell line 293T and A549 cells upon DENV infection. (Figure 1, C and D).

### *Mmp3* shows antiviral activity against DENV infection

**Figure 2:**
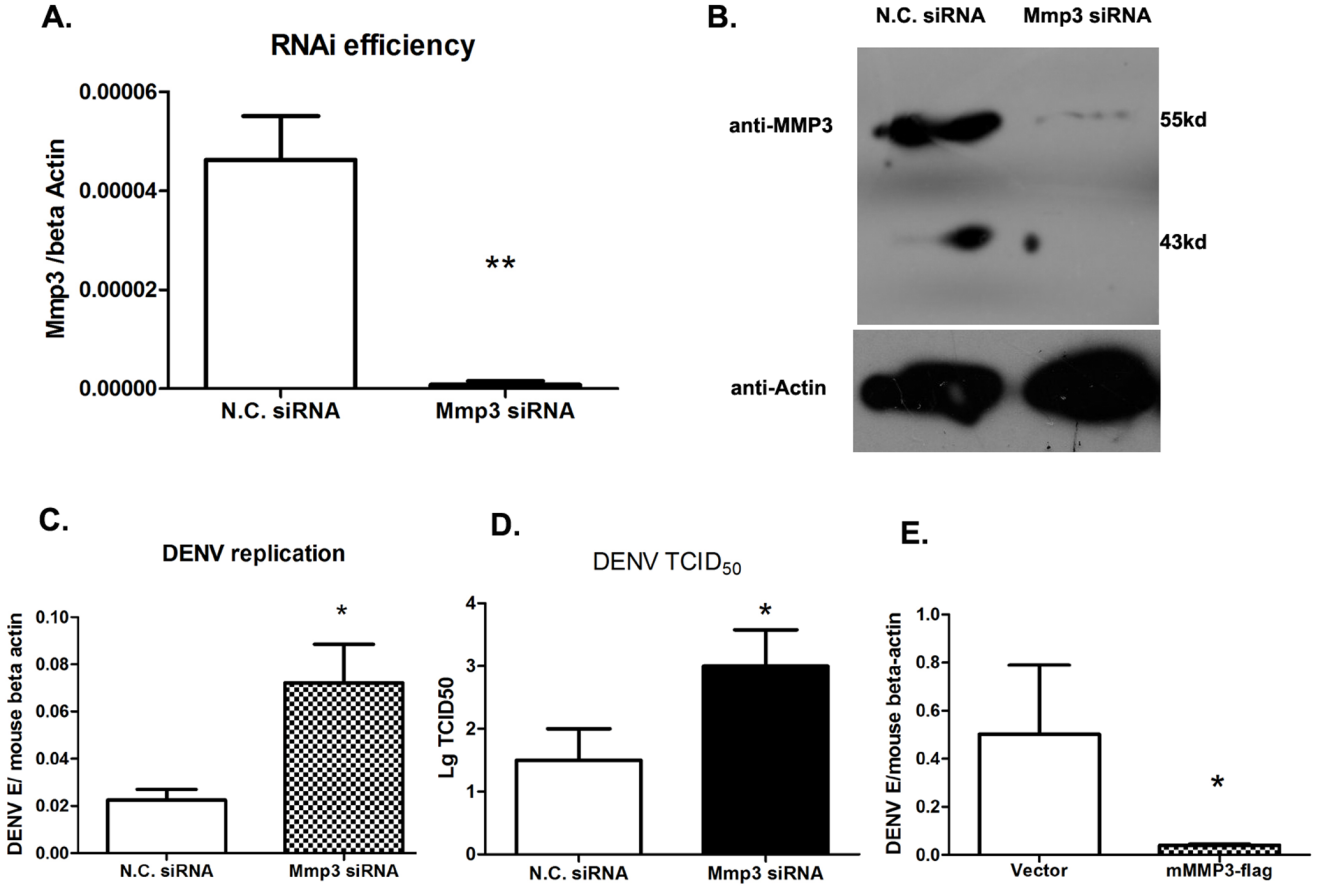
The antiviral effect of *Mmp3* against DENV. A-B) RNAi efficiency for *Mmp3* as shown in qRT-PCR (A) and Western blot (B). C-D) DENV burdens in RAW264.7 cells after RNAi silencing. C) The viral burdens were analyzed by measuring the virus E gene copy using qRT-PCR, and normalized to mouse beta actin gene. D) The titer of infectious DENV in cell supernatants 24 hrs post infection, as measured by TCID_50_ assay. E) The viral burdens in *Mmp3* overexpressed cells were decreased comparing with that in control cells. Results are expressed as the mean + the SEM. * p<0.05 and ** p<0.01(*t*-test). Representative results from at least 3 independent experiments.

To study the role of MMP3 during DENV infection, an siRNA based RNA interference study was performed in RAW264.7 cells. MMP3 was silenced efficiently as analyzed by qRT-PCR (Figure 2A) using gene specific primers and by Western Blot (Figure 2B). The intracellular viral loads, in terms of the transcript levels of the DENV envelop gene (E), was increased 2-fold (p<0.05) in MMP3 silenced cells compared with control cells (Figure 2C). To measure the production of infectious virus from these cells, a TCID_50_ assay was performed in Vero cells. The titers of virus in supernatants from MMP3 silenced cells were about 100-fold (2-log of 10) higher compared with that from control cells (Figure 2D). The anti-DENV role of MMP3 was further demonstrated in the murine *Mmp3* overexpressed RAW264.7 cells, in which the viral load was about 10-fold less than that of controls (Figure 2E). Taken together, our results indicated that MMP3 has an anti-viral activity against DENV replication in RAW264.7 cell.

### Cytokines are downregulated in *Mmp3* silenced cells upon DENV infection

**Figure 3:**
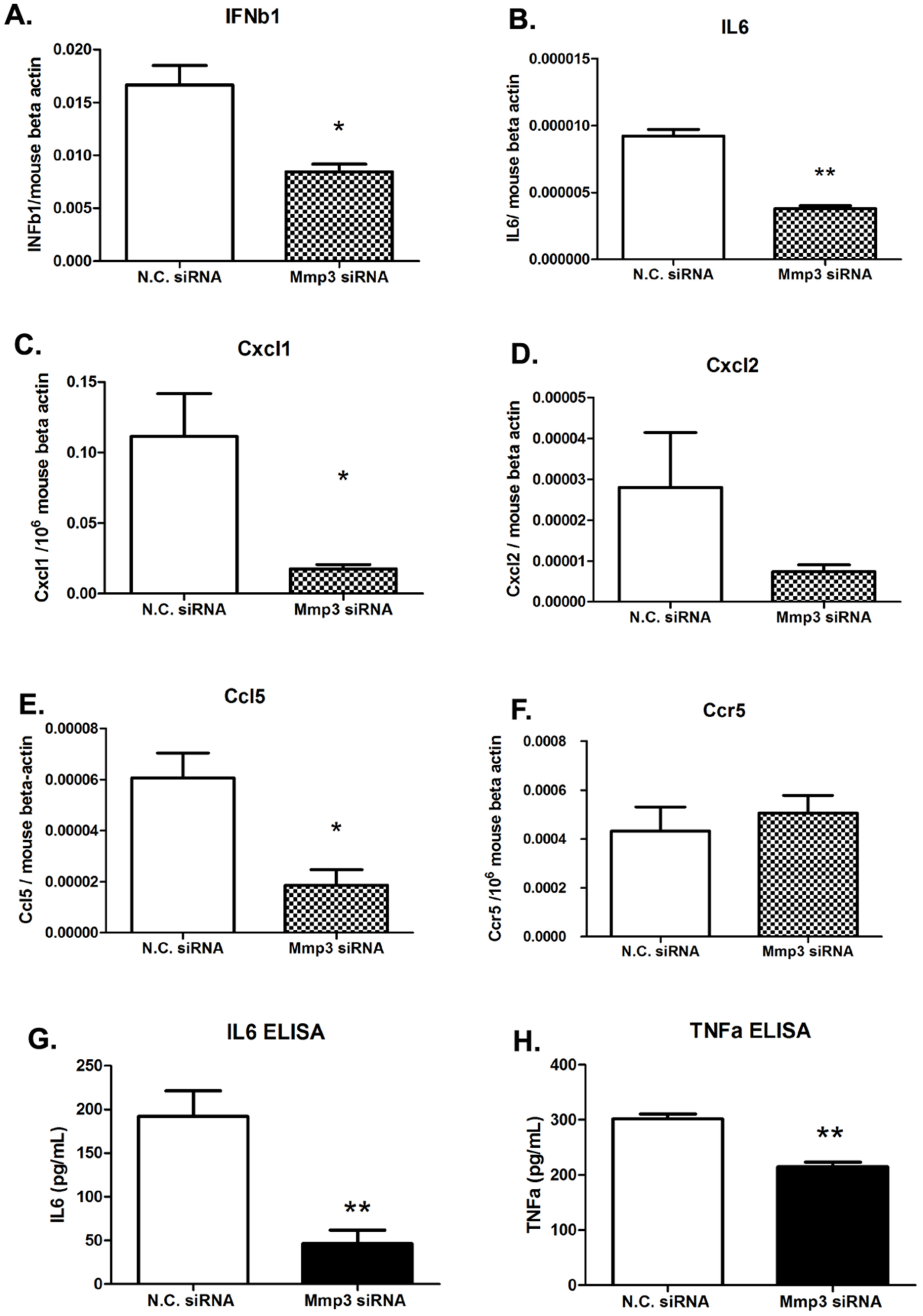
Cytokine expression in DENV infected RAW264.7 cells after gene silencing. A–F) mRNA levels of selective cytokines/chemokines (A: *Ifnβ1*, B: *Il 6*, C:*Cxcl1*,D:*Cxcl2*,E:*Ccl5*,F:*Ccr5*) were measured by qRT-PCR and normalized to mouse beta-actin gene. G–H) Protein level of TNFα and IL6 in cell supernatants. Results are expressed as the mean + the SEM. * p<0.05 and ** p<0.01. (*t*-test) Representative results from at least 3 independent experiments.

In order to investigate whether the knock-down of *Mmp3* would affect the other anti-viral cytokines during DENV infection, we compared the transcription levels of some cytokines and chemokines in *Mmp3* silenced cells by qRT-PCR. We found that the transcription levels were decreased about 3-fold for *Cxcl1* and *Ccl5* (p<0.05) and about 2-fold for *Ifnβ1* and *Il6* (p<0.05) in *Mmp3* silenced cells in comparison with control cells (Figure 3, A–F). The protein secretion of TNFα and IL6 were also decreased in *Mmp3* silenced cells comparing with controls (Figure 3, G and H). These data suggested that antiviral role of *Mmp3* may associate with its ability to modulate the production of some antiviral or pro-inflammatory cytokines during DENV infection.

### MMP3 is translocated from cytoplasm into cell nucleus and influences transcriptional factor activity of NFκB during DENV infection

**Figure 4:**
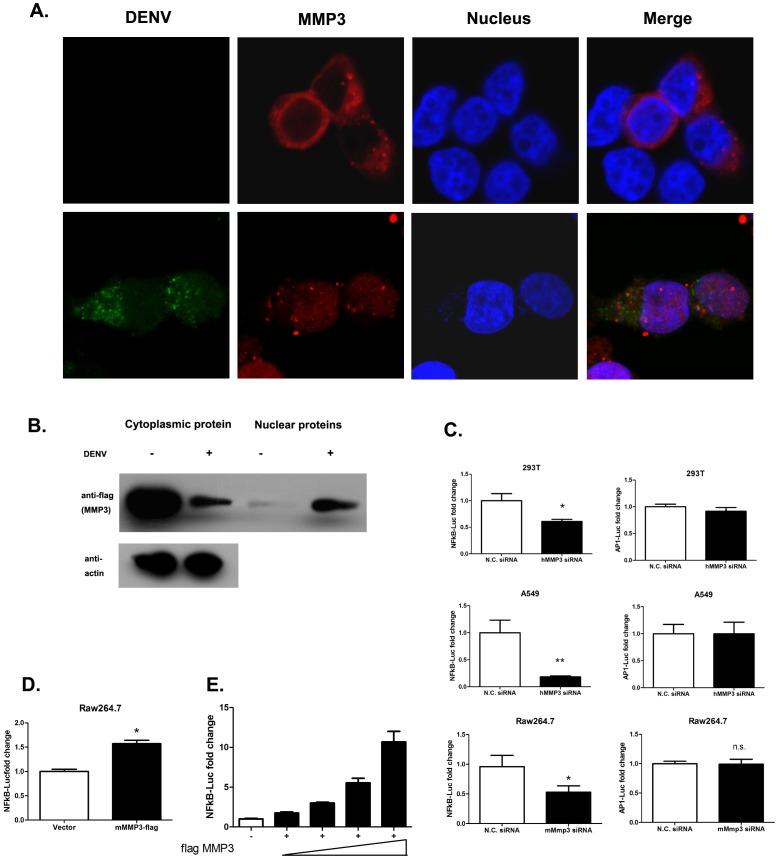
MMP3 moves from cell cytoplasm into nucleus upon DENV infection and influence NFκB activity. A) MMP3 moves from cell cytoplasm into nucleus upon DENV infection. B) The cytoplasmic and nuclear distribution of overexpressed flag-MMP3 in DENV infected or uninfected cells as determined by Western Blot. C)NFκB transcriptional activity is impaired upon MMP3 silencing in DENV infected 293T,A549 and RAW264.7 cells, while AP1 transcriptional activities were not influenced. D and E) Overexpression of MMP3 activates NFκB activity in DENV infected RAW264.7 (D) and 293T(E) cells. MMP3 activated NFκB in a dose dependent manner in DENV infected 293T cells(E) (MOI = 1.0, 24 hrs post infection). (The reporter activities were normalized by internal control (pRL-TK Renilla luciferase value). The mean value of activities from DENV infected control cells were set to 1.0) Results are expressed as the mean + the SEM. * p<0.05 and ** p<0.01 (*t*-test). Representative results from at least 3 independent experiments.

To further analyze the function of MMP3 during DENV infection, the subcellular distribution of MMP3 protein in DENV infected or uninfected cells were examined by confocal microscope. In uninfected cells, MMP3 predominantly distributed in cell cytoplasm as described previously[11]. While, Figure S1). This result was further confirmed by Western Blot using purified cytoplasmic or nuclear proteins from infected or uninfected cells. It clearly showed the significant increase of MMP3 protein in nuclear fraction in DENV infected cells (Figure 4B).

Since MMP3 were translocated into the nucleus upon DENV infection and influenced the cytokine production, we then investigated whether the MMP3 regulates activity of the related transcriptional factors. Both NFκB (Nuclear factor kappa-light-chain-enhancer of activated B cells) and AP1 (activator protein 1) play crucial roles in antiviral innate immune response through promoting the transcription of numerous antiviral or pro-inflammatory genes[24], [25]. Then, we compared the activities of NFκB and AP1 between MMP3 silenced cells and control cells that previously infected with DENV. The activities of NFκB and AP1 were monitored by the Luciferase reporter assay in RAW264.7, 293T and A549 cells, respectively. Our results showed that in comparison with controls, the NFκB activity was decreased by 50∼80% in MMP3 silenced cells; while the AP1 activity was not affected (Figure 4C).

To confirm the influence of MMP3 on NFκB activity, the effect of overexpression of MMP3 on NFκB activity was analyzed. As shown in Figure 4D and E, overexpression of MMP3 activated NFκB activity in both RAW264.7 and 293T cells during DENV infection. The activation of NFκB by overexpression of MMP3 was shown in a dose dependent manner in DENV infected 293T cells (Figure 4E). Furthermore, The expression of *Ifnα, Cxcl1* and *Cxcl2* were also upregulated in MMP3 overexpressed RAW264.7 cells after DENV infection when compared with control cells (Figure S2). These results suggested that upon DNEV infection, MMP3 could be translocated into cell nucleus and activate the NFκB, thereby promote the production of anti-viral or pro-inflammatory cytokines/chemokines.

### MMP3 interacts with NFκB in the nucleus of DENV infected cells

**Figure 5:**
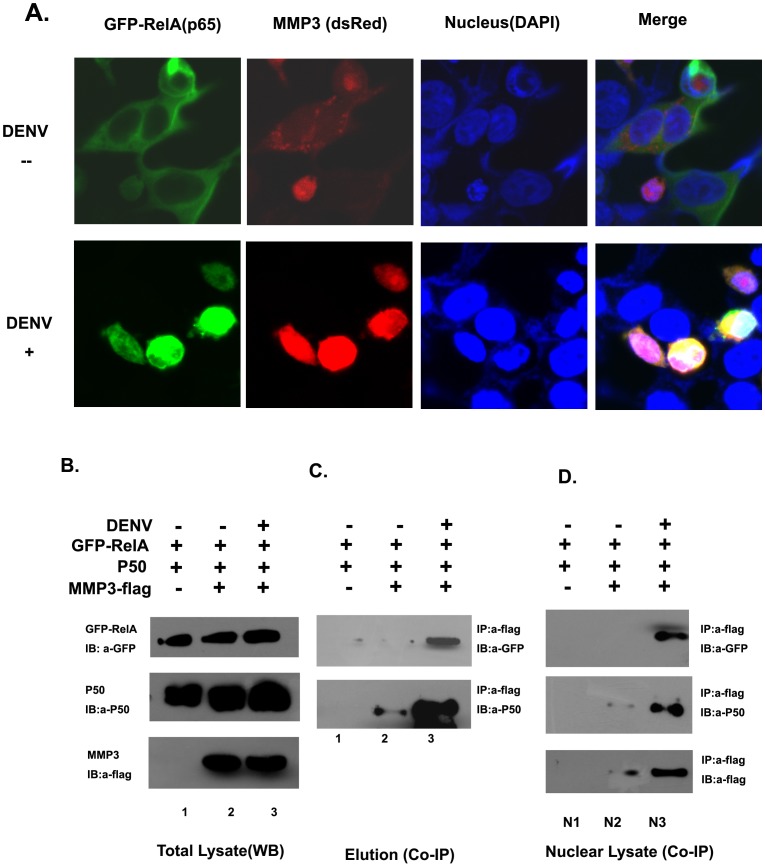
MMP3 interacts with NFκB in cell nucleus upon DENV infection. A) Co-localization of MMP3 and NFκB RelA(p65) in cell nucleus upon DENV infection. B and C) NFκB RelA (p65) and P50 were co-immunoprecipitated with MMP3 in total cell extract from DENV infected cells. The co-IP was carried out using anti-flag affinity gel and the bound proteins were detected by immunoblotting with anti-GFP, anti-P50 and anti-flag antibodies, respectively. D) NFκB RelA (p65) and P50 were co-immunoprecipitated with MMP3 in cell nucleus upon DENV infection. Representative results from at least 3 independent experiments.

Since MMP3 moved into cell nucleus and affected the activity of NFκB, we further explored the potential relationship between nuclear MMP3 and NFκB in DENV infected cells. Confocal microscopy showed that MMP3 co-localized with NFκB RelA (p65) in DENV infected cell nucleus. While in uninfected cells, these two proteins were randomly distributed in cell cytoplasm (Figure 5A).

The colocalization of MMP3 and NFκB RelA (p65) suggested the possibility of interaction between the two proteins during DENV interaction. Thus, a co-immunoprecipitation (co-IP) was performed to investigate the potential interaction of MMP3 and NFκB in cells co-transfected with MMP3, RelA and P50 (Figure 5, B and C). The result showed that NFκB RelA(p65) and P50 were co-immunoprecipitated with MMP3 in DENV infected cells (Figure 5C). To further analyze whether MMP3 can interact with NFκB in cell nucleus, the nuclear extract of transfected cells were isolated and a co-IP assay were performed using the same protocol. Indeed, being consistent with the result using total cell lysate, the MMP3 co-immuprecipitated NFκB RelA(p65) and P50 in nucleus of DENV infected cells (Figure 5D). Our data suggested that MMP3 is translocated into nucleus upon DENV infection, interacts with and activates NFκB, thereby promotes the production of anti-viral cytokines.

### The MMP3 C-terminal hinge and hemopexin-like domain is involved in interaction with NFκB in DENV infected cells

**Figure 6:**
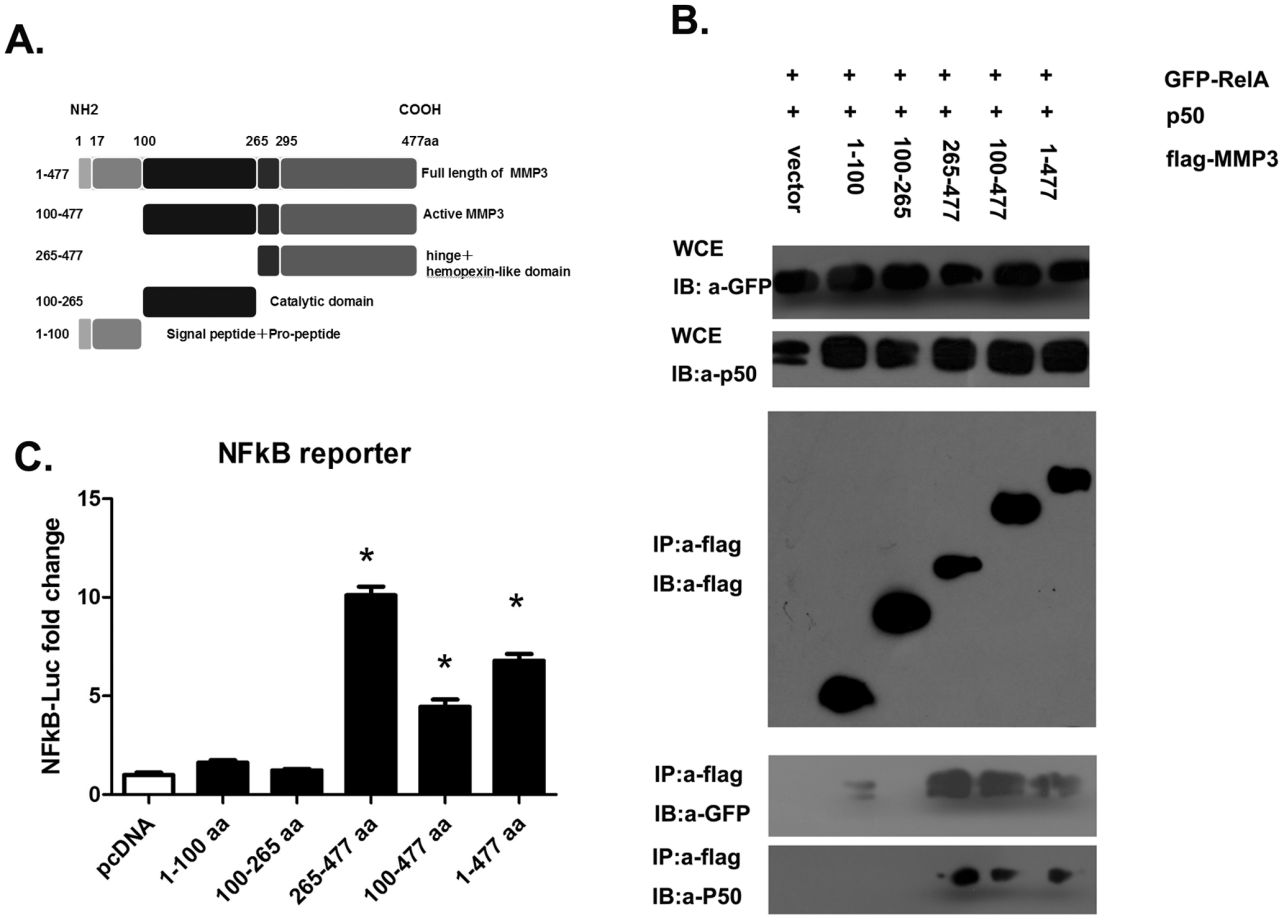
The C-terminal hinge and hemopexin-like domain of MMP3 is required for interaction with NFκB. A) Domain structure of MMP3 and the truncated MMP3 used in this study. B) Co-immunoprecipitation assay with various domains of MMP3 and NFκB (RelA(p65) and P50). C) Influence of NFκB activity by various domains of MMP3 measured by Luciferase reporter assays. Results are expressed as the mean + the SEM. * p<0.05, when compared with controls(*t*-test). Representative results from at least 3 independent experiments.

MMP3 is composed of five structural and functional domains[26] (Figure 6A). To further identify the domains involved in the interaction with NFκB, we performed Co-IP with either the full length or the truncated MMP3 proteins. As shown in Figure 5B, the NFκB RelA (p65) and P50 were co-immunoprecipitated with the full length and two truncated MMP3, i.e. 100–477aa and 265–477aa that contain the hinge and hemopexin-like domain (Figure 6B, lanes 4 to 6). There was only a faint GFP-RelA band visible in Co-IP, but no P50 was pulled-down in lane 2 (1–100aa). Perhaps, the prepeptide domain (1–100aa) has weak interaction with NFκB RelA (p65) and it requires further investigation. No NFκB was co-precipitated with the MMP3 catalytic domain (100–265aa) (lane 3). These results indicated that the C-terminal hinge and hemopexin-like domain are required for the interaction with NFκB, while the leading sequence (1–100aa) and the catalytic domain (100–265aa) are not necessary.

To confirm this result, a NFκB luciferase reporter assay were performed in cells expressing these truncated MMP3 proteins. In line with the results from the co-IP, only fragments containing C-terminal hinge and hemopexin-like domain (265–477aa, 100–477aa, and 1–477aa) had the ability to activate NFκB (Figure 6C). These results further confirmed that MMP3 interacts with NFκB via its C-terminal hinge and hemopexin-like domain and activates NFκB upon DENV infection, thereby exerts an antiviral effect by promoting the NFκB-directed cytokine production.

## Discussion

Traditionally, MMPs are considered as zinc-dependent endopeptidases that are involved in the remodeling and turnover of the ECM in physiological or pathological states. However, recent findings indicate that matrix metalloproteinases act on pro-inflammatory cytokines, chemokines and other proteins to regulate varied aspects of inflammation and immunity[12]. Of note, MMPs are well known as secretory endopeptidases; but accumulating evidence suggested the presence of MMPs in the cell nucleus, implicating the new role of this old family[27]. In addition to MMP3[26], [28], other MMPs including MMP2[29], MMP13[30], MT1-MMP[31], and ADAMTS13[32] were also found in cell nucleus under different conditions. Recent report suggested that there are 6 putative nuclear localization signals in the primary sequence of MMP3, and these NLS are responsible for the nuclear translocation of MMP3 [26]. However, very little is known about the distinct function of MMP3 in nucleus. Si-Tayeb K *et al.* suggested that MMP3 is present in nucleus and related with apoptosis[28]. Eguchi T *et al.* indicated a transcriptional factor like activity of MMP3 in cell nucleus in chondrocytes. MMP3 binds to transcription enhancers in the connective tissue growth factor gene (CTGF/CCN2)[26]. Further, this study demonstrated that MMP3 might interact with some proteins in cell nucleus including HP1γ,NF45,NCOR1 and RBBP4[26].

In our present study, we indicated that MMP3 was translocated into the cell nucleus upon virus infection. Additionally, MMP3 was co-localized with NFκB and interacted with NFκB complex via its C-terminal hinge and hemopexin-like domain in DENV infected cells. This interaction promotes the anti-viral cytokine production directed by NFκB. Interestingly, in Eguchi T's study, MMP3 was suggested to interact with NCoR1[26], a well known constitute co-suppressor of NFκB[24], [33]. We have once hypothesized that MMP3 might interact with NCoR1 and NFκB at the same time, and then remove the suppression of NCoR1 from NFκB. But our co-IP experiments can only fish out NFκB p65 and p50, but not NCoR1, using MMP3 as a bait (data not shown). This suggested that MMP3 may directly interact with NFκB and activate it in a NCoR-independent manner.

In addition to JAK-STAT pathway, the mRNA expression of MMP3 is also directly modulated by the transcription factor NFκB[34]. And here we showed that during virus infection, MMP3 protein level was evaluated, and MMP3 was translocated into cell nucleus and interacted with and further activated NFκB. This forms a positive feedback between MMP3 expression and NFκB activation, and may amplify the antiviral response in cells. Furthermore, we have found that MMP3 does not influence the degradation of IκB, the major upstream factor of NFκB (data not shown). Woo MS *et al.* reported that during LPS stimulation, the inhibitor against MMP3 could inhibit the ability of NFκB binding to its responsive DNA elements[21]. Li C *et al.* showed that immunity against intestinal bacterial infection is impaired in MMP3 deficient mice, and the TNFα level was lower in these knockout mice compared with wild type during early infection [35]. All these findings suggest that MMP3 may have a role on modulation of NFκB mediated inflammatory response under various condition of infection. To confirm this, luciferase reporter assay was carried out in cells treated with LPS or Poly I:C. The NFκB activity was significantly decreased in MMP3 silenced cells compare with controls upon both stimulations (Figure S3).

Taken together, our finding suggested a novel role of an old MMP in cell nucleus during DENV infection, and provided new target for the control of DENV infection based on modulation of MMP3 activity.

## Supporting Information

Figure S1 **MMP3 presented in cell nucleus upon DENV infection in RAW264.7 cells.** ***Endogenous MMP3 were labeled with anti-MMP3 antibody and detected with FITC labeled secondary antibodies under confocal microscope. Arrows indicate MMP3 presents in cell nucleus upon DENV infection.***
(TIF)

Figure S2 **Cytokine and chemokine expression were upregulated in MMP3 overexpressed RAW264.7 cells upon DENV infection.** A) Relative *Mmp3* mRNA level in *Mmp3* overexpressed cells compared with control cells. B–D) mRNA level of *Ifnα*, *Cxcl1* and *Cxcl2* increased in *Mmp3* overexpressed RAW264.7 cells upon DENV infection. Gene expression were measured by qRT-PCR and normalized to mouse beta-actin gene. Results are expressed as the mean + the SEM. * p<0.05 and ** p<0.01 (*t*-test). Representative results from at least 3 independent experiments.
(TIF)

Figure S3 **NFκB activity was impaired in MMP3 silenced cells upon stimulation with LPS or Poly I:C.** A, C) NFκB luciferase activity in 293T cells treated with LPS (A) or Poly I:C(C). B, D) AP1 luciferase activity in 293T cells treated with LPS (B) or Poly I:C(D). The mean value of activities from control cells were set to 1.0. Results are expressed as the mean + the SEM. * p<0.05(*t*-test). Representative results from at least 3 independent experiments.
(TIF)
