# Supplementary figures and images for: Matrix Metalloproteinase 3 Promotes Cellular Anti-Dengue Virus Response via Interaction with Transcription Factor NFκB in Cell Nucleus
Source: PLoS One. 2014 Jan 8;9(1):e84748. doi: 10.1371/journal.pone.0084748 (PMC3885614; doi:10.1371/journal.pone.0084748)

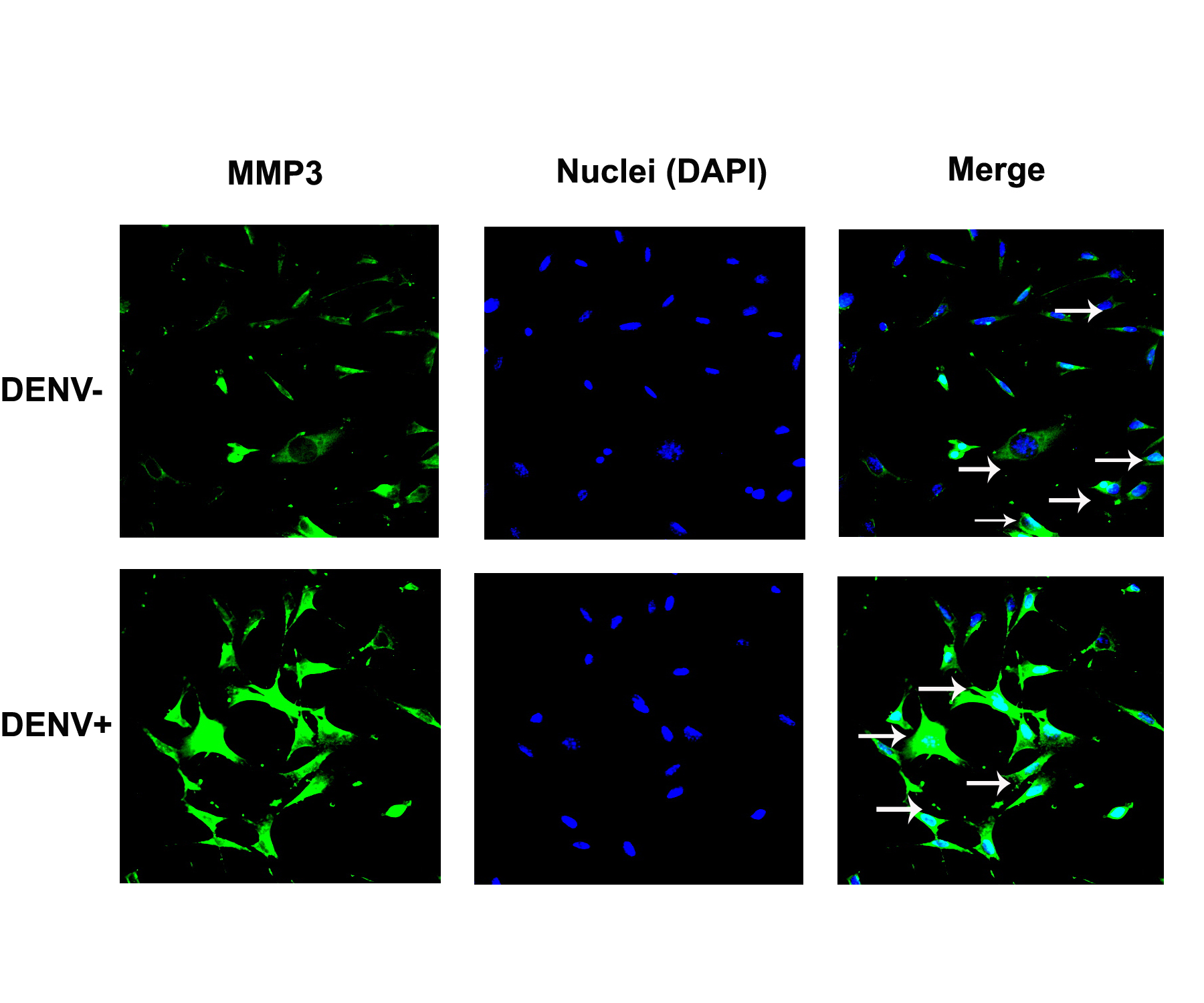

Supplement: Figure S1 — MMP3 presented in cell nucleus upon DENV infection in RAW264.7 cells. Endogenous MMP3 were labeled with anti-MMP3 antibody and detected with FITC labeled secondary antibodies under confocal microscope. Arrows indicate MMP3 presents in cell nucleus upon DENV infection. (TIF) [file pone.0084748.s001.tif]

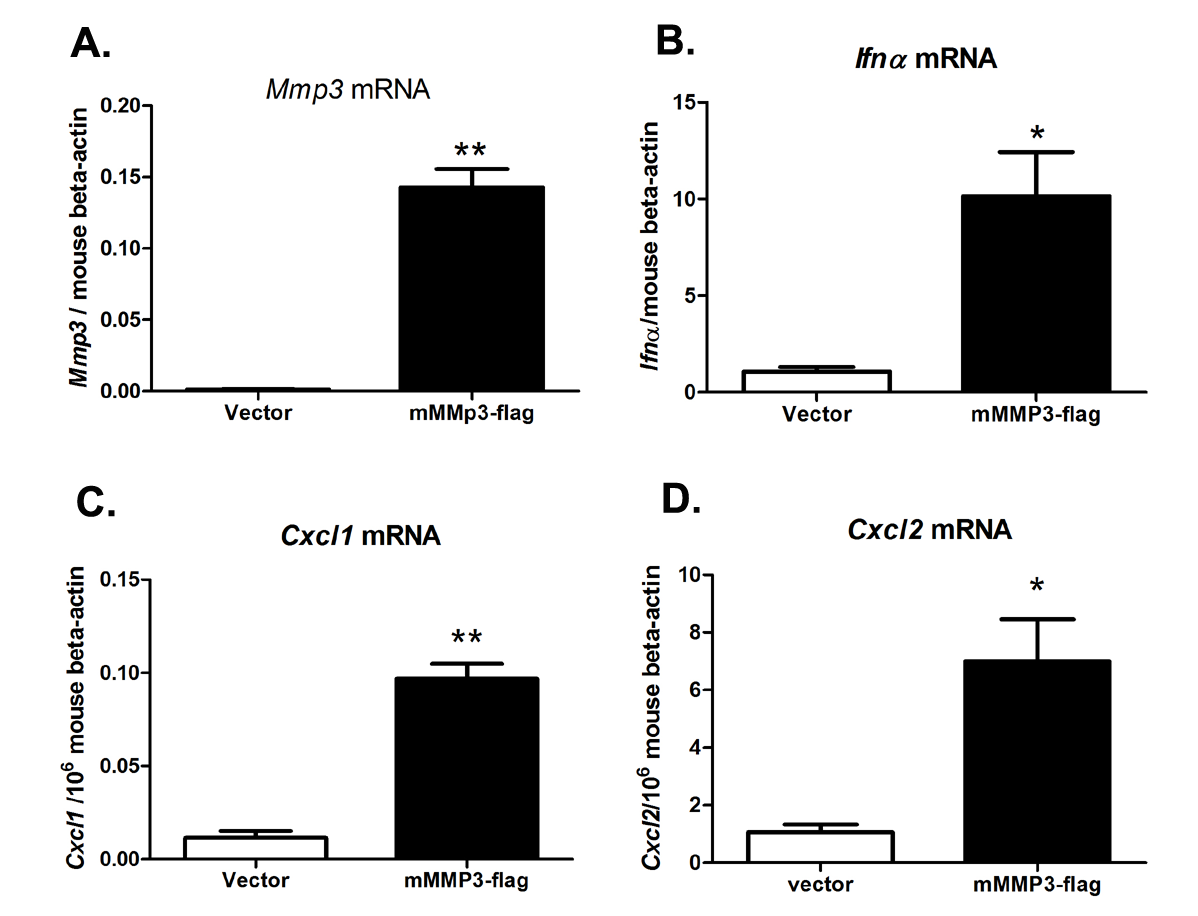

Supplement: Figure S2 — Cytokine and chemokine expression were upregulated in MMP3 overexpressed RAW264.7 cells upon DENV infection. A) Relative Mmp3 mRNA level in Mmp3 overexpressed cells compared with control cells. B–D) mRNA level of Ifnα, Cxcl1 and Cxcl2 increased in Mmp3 overexpressed RAW264.7 cells upon DENV infection. Gene expression were measured by qRT-PCR and normalized to mouse beta-actin gene. Results are expressed as the mean + the SEM. * p<0.05 and ** p<0.01 (t-test). Representative results from at least 3 independent experiments. (TIF) [file pone.0084748.s002.tif]

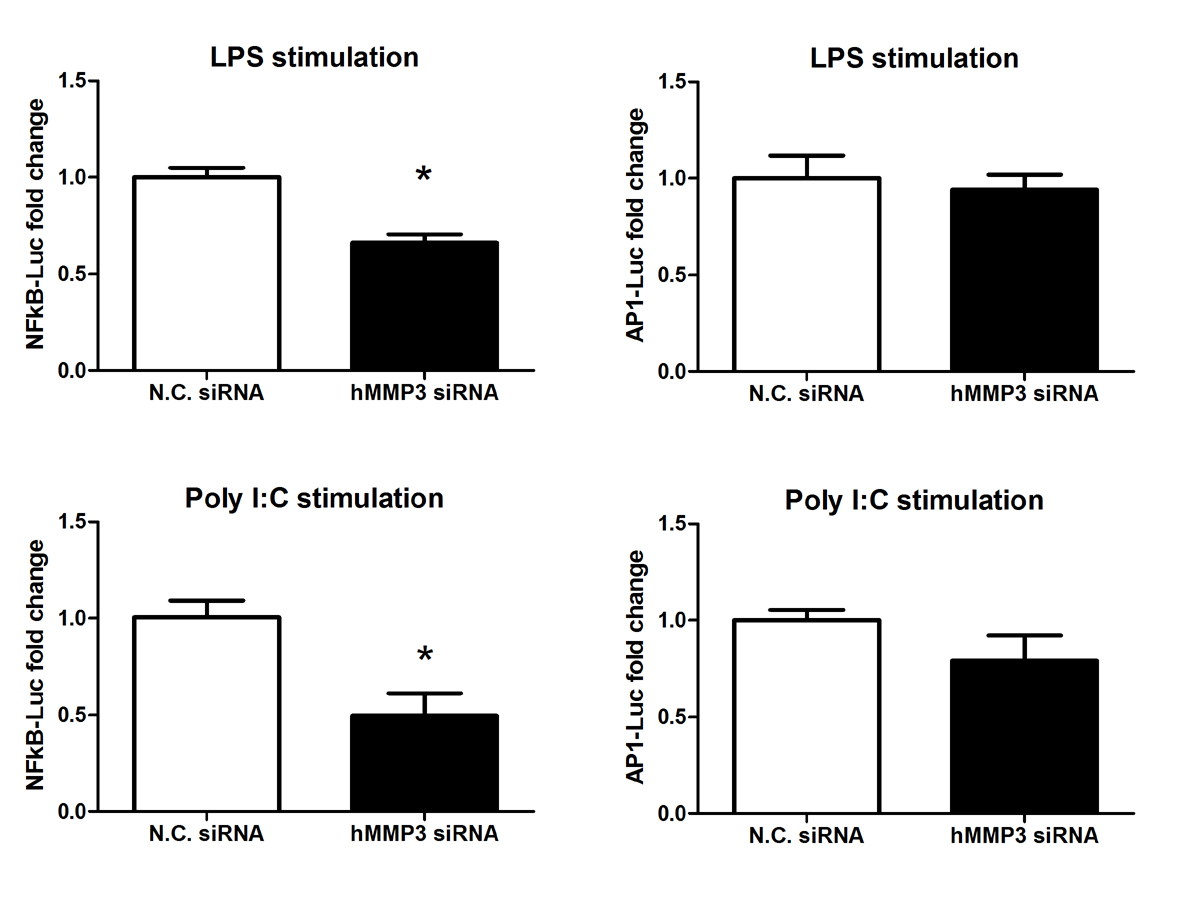

Supplement: Figure S3 — NFκB activity was impaired in MMP3 silenced cells upon stimulation with LPS or Poly I:C. A, C) NFκB luciferase activity in 293T cells treated with LPS (A) or Poly I:C(C). B, D) AP1 luciferase activity in 293T cells treated with LPS (B) or Poly I:C(D). The mean value of activities from control cells were set to 1.0. Results are expressed as the mean + the SEM. * p<0.05(t-test). Representative results from at least 3 independent experiments. (TIF) [file pone.0084748.s003.tif]
